# Supplementary material for: Inhibition of Melanoma Cell Migration and Invasion Targeting the Hypoxic Tumor Associated CAXII
Source: Cancers (Basel). 2020 Oct 17;12(10):3018. doi: 10.3390/cancers12103018 (PMC7602957; doi:10.3390/cancers12103018)
Supplement: Supplementary file 1 [file cancers-12-03018-s001.pdf]

Supplementary Materials: Inhibition of Melanoma Cell Migration and Invasion Targeting the Hypoxic Tumor Associated CAXII

Gaia Giuntini, Sara Monaci, Ylenia Cau, Mattia Mori, Antonella Naldini and Fabio Carraro

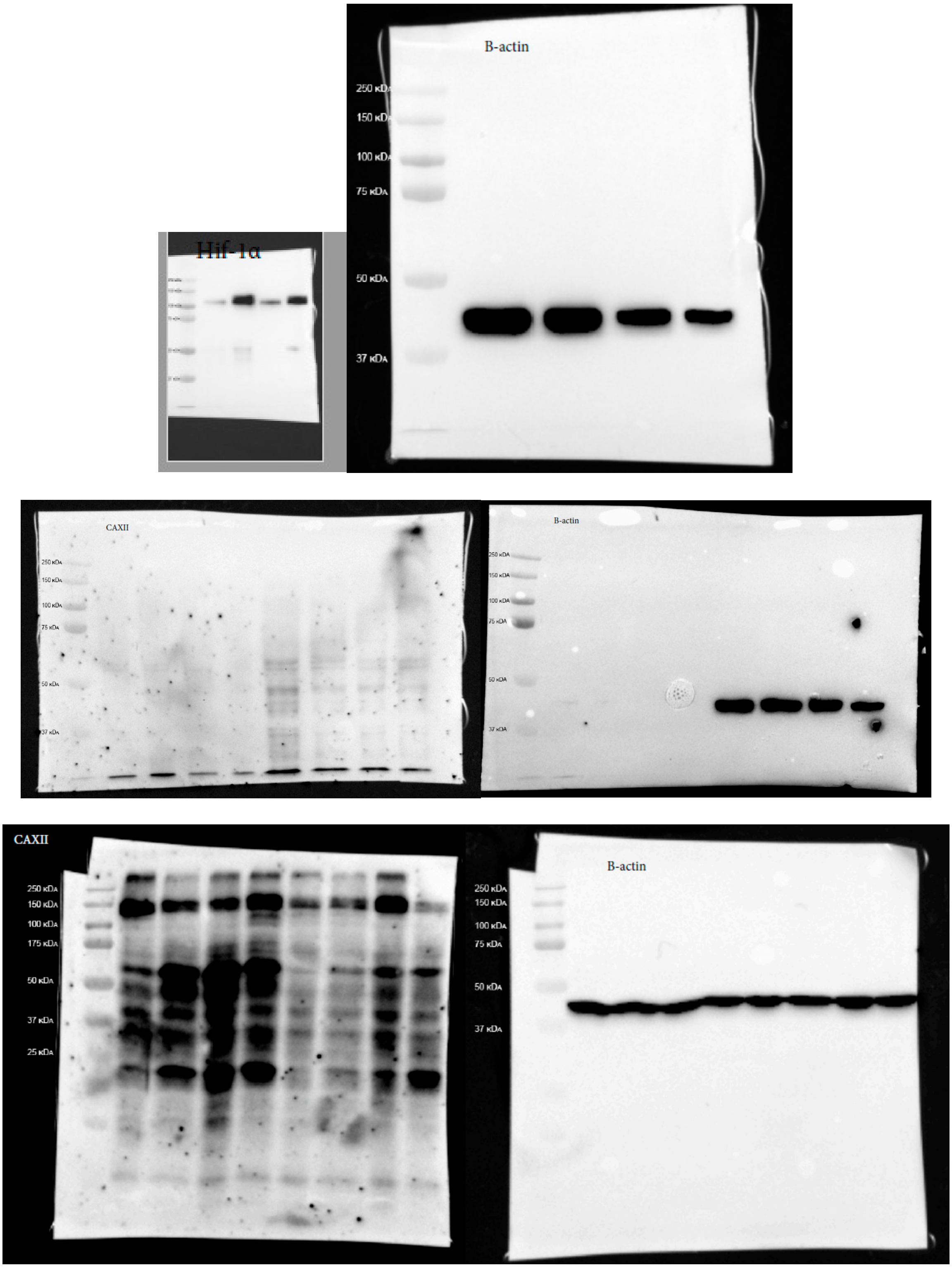

Figure S1. The uncropped blots of Figure 1A,D.

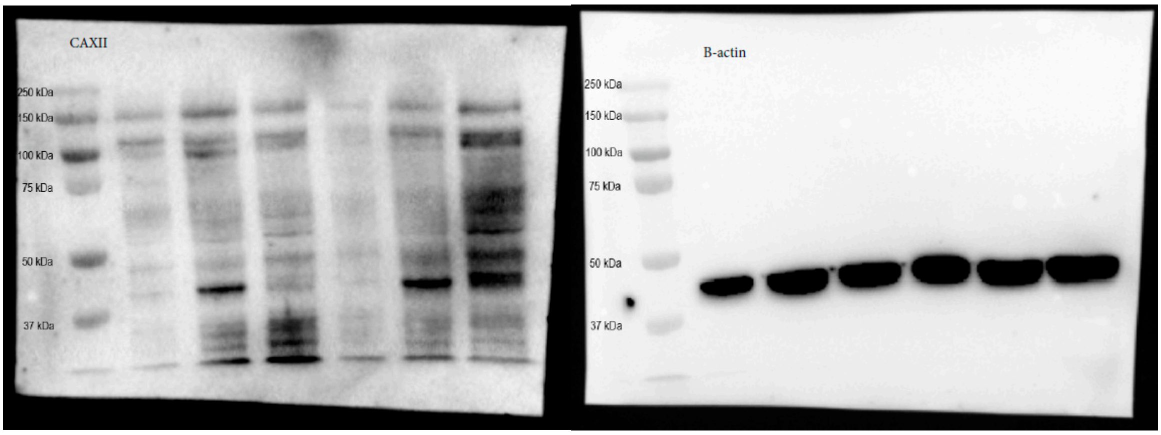

Figure S2. The uncropped blots of Figure 2A.

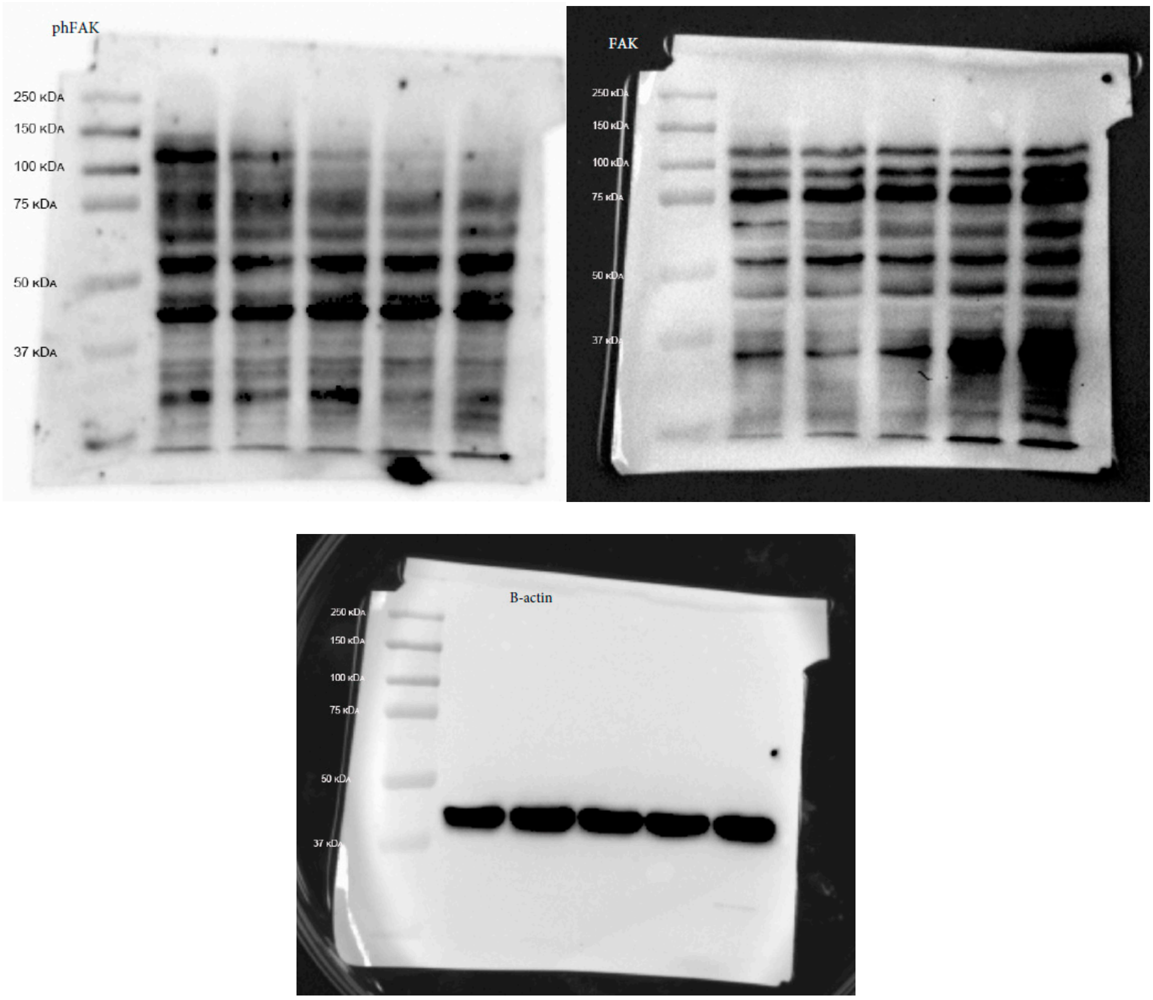

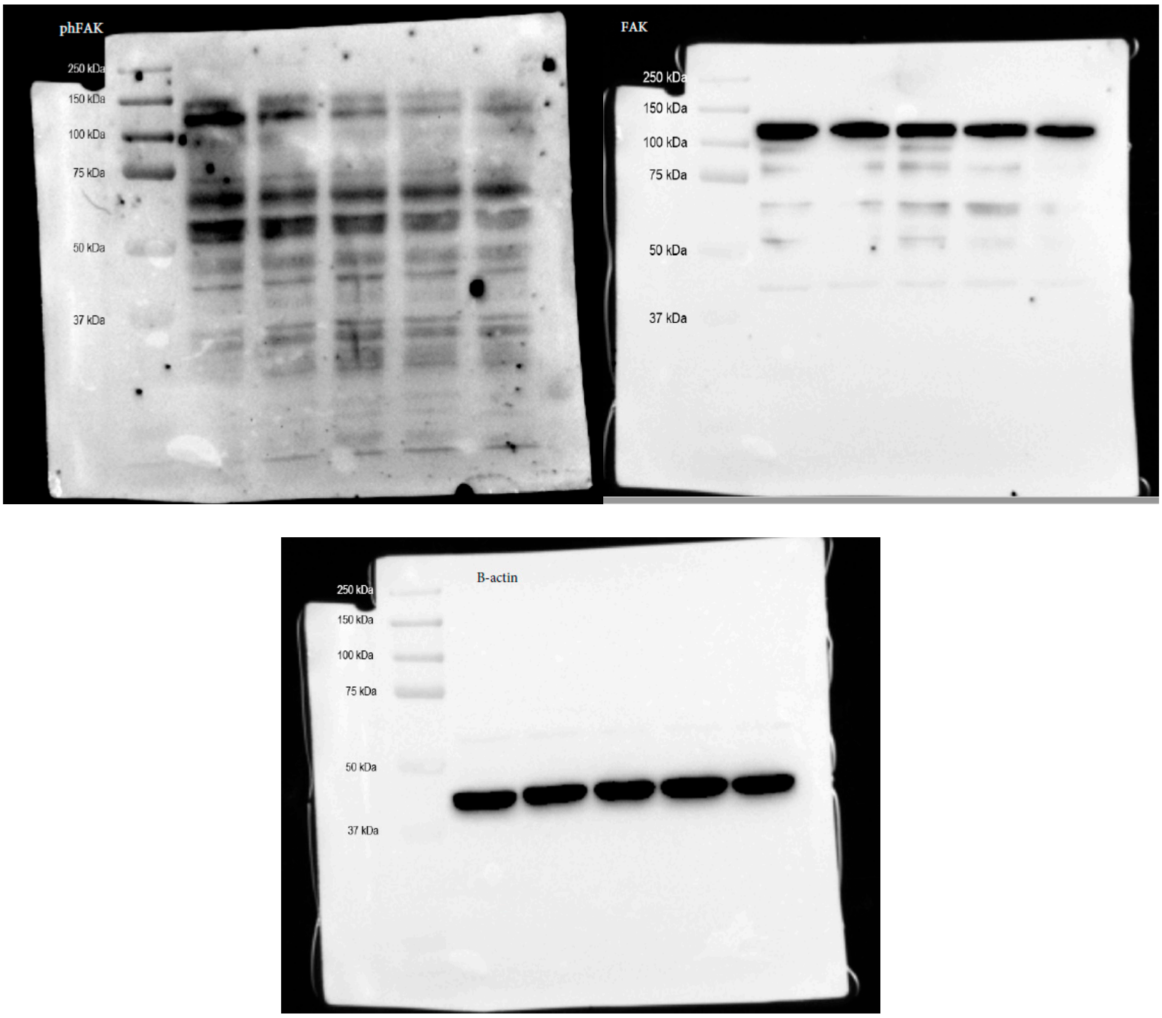

Figure S3. The uncropped blots of Figure 3B.

© 2020 by the authors. Licensee MDPI, Basel, Switzerland. This article is an open access article distributed under the terms and conditions of the Creative Commons Attribution (CC BY) license (http://creativecommons.org/licenses/by/4.0/).
